# Predictors of mental health help-seeking among polish people living the United Kingdom

**DOI:** 10.1186/s12913-018-3504-0

**Published:** 2018-09-06

**Authors:** Dawid Gondek, James B. Kirkbride

**Affiliations:** 1 Centre for Longitudinal Studies, UCL Institute of Education, 55-59 Gordon Square, London, WC1H 0NU UK; 2 Division of Psychiatry, UCL, London, W1T 7NF UK

**Keywords:** Access, Help-seeking, Mental health, Migration, United Kingdom

## Abstract

**Background:**

Migration has been shown to be associated with negative mental health outcomes. Moreover, migrants tend to underutilise mental health services. The current study aimed to assess the association between predictors, divided into three groups (predisposing, enabling and need), and two outcome variables: (1) past professional mental health help-seeking during the stay in the United Kingdom; (2) intentions of mental health help-seeking from a mental health professional within the next three months.

**Methods:**

The study utilised a population-based cross-sectional survey with the final sample of 536 participants. Multivariate linear and logistic regression models were used to examine the association between predictors and the outcomes.

**Results:**

We found strong evidence that older age, mental health stigma and living circumstances (predisposing factors), as well as knowledge of the National Health Service, social support, and education (enabling factors) were associated with past and future help-seeking for mental health problems. Finally, mental health status was associated with both past help-seeking and intentions.

**Conclusion:**

Due to large numbers of migrants in the UK it is vital to ensure that these populations receive adequate mental health support. Findings of the present study may inform development of policies and interventions better tailored to specific migrant populations.

**Electronic supplementary material:**

The online version of this article (10.1186/s12913-018-3504-0) contains supplementary material, which is available to authorized users.

## Background

The Polish-born population in the United Kingdom increased from 58,000 in 2001 to 679,000 in 2013, with Poles becoming the second-largest foreign group in the country [1]. The Belfast Health and Social Care Trust identified Polish migrants as being at higher risk of depression, substance abuse and suicide compared with other migrant groups [2]. Polish migrants and other Eastern Europeans exhibit particularly aggressive and destructive drinking behaviours – with excessive alcohol consumption being a potential cause and consequence of depression [3, 4]. Furthermore, Polish nationals, among other Eastern Europeans, constitute the greatest proportion of homeless individuals in the UK – with homelessness being associated with substance abuse and mental health problems [5]. Hence, there is a clear need for robust evidence on help-seeking behaviours among Polish migrants, which can facilitate early detection of mental health problems. We believe that findings obtained with Polish population can be generalised to other Easter European communities due to similar social, cultural and demographic characteristics. Currently, there is a dearth of evidence regarding mental health status and help-seeking behaviours among this population. Available findings rely on a few small-scale, mainly qualitative studies that were published in non-peer-reviewed format – making it difficult to determine their quality [6–10].

More generally, there is robust evidence demonstrating that migrant populations, particularly members of ethnic minorities, are at greater risk of developing certain mental health problems, particularly psychotic disorders [11, 12]. This risk may extend to European migrant groups [13, 14], including people of Eastern European origin. As far as Polish, or Eastern European, migrants are concerned, the evidence is limited to small-scale, mainly non-peer-reviewed studies. For example, Smolen conducted a population-based survey – with a sample of 286 Polish-born adults living in the UK for at least 12 months – showing that 36% were under permanent stress [8, 9]. While it remains unclear how this figure compares with that in the general population, due to a lack of comparable data, qualitative research has highlighted that migrant groups cite immigration as a stressful life event commonly contributing to a subsequent deterioration of their mental health [10].

There is also evidence that migrants, including the Polish population in the UK, underutilise mental health services [6, 7, 15]. For example, Smolen found that only 5% of Polish respondents, out of 286, consulted a psychologist, while only 1% consulted a psychiatrist, despite the fact that 42% of respondents reported long term high levels of stress [8]. Reasons for underutilisation of services include limited knowledge and trust of the National Health Service (NHS), previous negative experience, poor language skills and perceived cultural differences related to how migrants describe their mental health problems [6, 7, 16]. Moreover, mental health stigma appears to be a serious barrier to accessing mental health services, particularly among those with untreated mental health problems, which may contribute to greater weight being placed on self- or family-reliance, further contributing to reluctance to seek professional mental health support [7, 16].

In order to effectively help Polish and other European migrants to address their mental health needs, more research is needed to fully understand factors predicting help-seeking behaviours and intentions. Here, we sought to identify the characteristics of Polish migrants in the UK that facilitate and impede utilisation of mental health services. Our study was based on a theoretical model of health service usage, first proposed by Andersen and Newman [17]. The model posits that service use is dependent on three core groups of characteristics: (1) demographic and social characteristics (predisposing factors); (2) ability to access services (enabling factors); (3) health problems leading to the need for health care services (need factors), either as perceived, or determined by objective measures [17]. We used this model, along with previous literature on help-seeking behaviours to guide data collection [7, 16, 18, 19]. We hypothesised that predisposing, enabling and need factors (see Table 1) would be independently associated with both past help-seeking behaviours and intentions of future help-seeking, and that the associations would remain significant after controlling for important confounders.

**Table 1:** Exposure variables hypothesised to be associated with greater intentions of help-seeking and higher odds of past help-seeking behaviours

Greater intentions of future help-seeking will be associated with:	Higher odds of past help-seeking behaviours will be associated with:
*- predisposing factors*:	*- predisposing factors*:
(1) being female	(1) being female
(2) being older	(2) being older
(3) being married	(3) being married
(4) longer length of stay in the UK	(4) longer length of stay in the UK
(5) presence of a partner in the UK	(5) presence of a partner in the UK
(6) presence of a child/children in the UK	(6) presence of a child/children in the UK
(7) lower help-seeking self-stigma	(7) lower help-seeking self-stigma
*- enabling factors*:	*- enabling factors*:
(1) having a degree	(1) having a degree
(2) being employed	(2) being employed
(3) greater perceived social support	(3) greater perceived social support
(4) better proficiency in English language	(4) better proficiency in English language
(5) better knowledge of NHS	(5) better knowledge of NHS
(6) greater acculturation	(6) greater acculturation
	(7) history of help-seeking behaviours
*- need factors*:	*- need factors*:
(1) poorer mental health	(1) poorer mental health

## Methods

### Sampling procedure

We conducted a cross-sectional, population-based survey, which was granted ethical approval by the University College London Research Ethics Committee (7151/001). The population of interest were Polish adults living in the United Kingdom, estimated to be 679,000 [1]. Recruitment took place from 17/08/15 to 24/11/15, using a combination of non-probability convenience and snowball sampling strategies. All participants were recruited online through Facebook and online Polish news media (Londynek.net; Emito.net) – in an attempt to reach those who do not use social media. Participants were also encouraged to share the information about the study with eligible friends and family.

First, we aimed to identify all Facebook groups primarily created for Polish communities in the UK. We entered key words into Facebook ‘search’ for three main types of groups: (1) people living in the same area – we entered ‘Polish people in’ (and Polish language equivalent) and the name of every city in the UK (*n* = 69; e.g. London, Manchester); (2) individuals sharing the same occupation – we entered ‘Polish’ and the name of different professions known to be common among Polish people (e.g. jobs in the construction industry, and allied trades), with Polish-language equivalents also used; (3) Polish student societies – we entered ‘Polish students at’, or the Polish equivalent, and the name of 30 different universities (top 30 universities from the Guardian University league table as more prestigious universities were more likely to have societies for Polish students) [20]. In addition, Facebook automatically recommended other potentially relevant groups based on our searches. After obtaining membership to those groups, we were able to access 76 groups created for people living in the same area (e.g., Polish people in London), two groups for individuals sharing the same occupation (e.g., road haulage occupations), seven groups for student societies (e.g., Edinburgh University Polish Society) and three other miscellaneous groups (Polish mothers, Eastern Europeans, Polish Professionals). The groups (*n* = 88) comprised the total of 31,4916 members at the end of data collection (24/11/15). However, this number has fluctuated during the data collection. In addition, it is unclear how many members were active users and how many individuals were members of more than one group. We excluded 50 (7.5%) participants who only completed demographic data, 79 (11.2%) participants with missing data on more than 40% of items and seven participants with no information on outcome data, resulting in a final sample of 536.

After receiving access to aforementioned groups, we posted the link to the survey launched with SurveyMonkey along with brief information explaining the purpose of the study and the inclusion criteria (first generation Polish individuals currently living in the UK, aged 18 years and older). Participants gave informed consent to take part in the online survey after having read the information page. To increase response rates, we offered an opportunity to take part in a prize draw to win one of three shopping vouchers worth between £20 and £50. The draw took place after collection of the data using an online random number generator.

### Instruments and measures

We used a set of instruments to elicit questions about sociodemographic characteristics, past mental health help-seeking behaviours and future intentions, and predisposing, enabling and need factors related to help-seeking (Additional file 1: Table S1). Where available, Polish versions of these instruments were obtained from the authors along with permission to use them. When unavailable, we translated them into Polish, with back-translation conducted based on established guidelines [21]: two independent forward translations were developed by bilingual translators (the author and a Polish psychologist, SC), and compared for any discrepancies in wording to create a common version of the instrument. Subsequently, a third bilingual person, with a profession not related to psychology or mental health (LL), blind to the original English version, back-translated the survey into English. This was compared with the original questionnaire to ensure validity. The process was planned to be repeated until the original and translated English versions were compatible, but this was achieved in the first cycle.

#### Outcome measures

We asked about intentions of future mental health help-seeking using the General Help Seeking Questionnaire (GHSQ) – a tool developed to capture help-seeking for mental health problems from both formal (e.g. mental health services) and informal (e.g. a friend) sources [22]. Previous research found the link between intentions of mental health help-seeking and subsequent actual help-seeking – establishing the predictive and construct validity of the measure [22].

Participants were asked to rate, using a 7-point Likert (from ‘extremely unlikely’ to ‘extremely likely’), the possibility of seeking help in next three months from five separate groups of professionals or organisations for emotional problems: (a) mental health professionals (e.g. counsellor, psychologist, psychiatrist), (b) phone help line (e.g. Samaritans), (c) family doctor/General Practitioner (GP), (d) charities, (e) other, not listed, professionals. The questions about informal help sources were not included as it was beyond the scope of the study. The authors of the measure advocated using both indicators of specific help sources and a summary score capturing an overall help-seeking intention [23]. As we were interested in finding predictors of help-seeking from any formal source – and the measure had a high internal consistency (Cronbach’s alpha = 0.80; Additional file 1: Table S1) – we used a total score ranging from 5 to 35, with higher score indicating greater intention to seek help in the future.

We also asked about past professional help-seeking behaviours for mental health problems using the GHSQ [22]. This was assessed using a binary item: ‘Have you ever seen a mental health professional in the UK (e.g. counsellor, psychologist, psychiatrist) to get help for personal problems?’. Although items on past help-seeking behaviours were primary outcomes of our study, we also considered them as predictors of intentions to seek help in the future.

#### Predictors

We collected data on demographic factors, knowledge of the mental health system in the UK, language proficiency, perceived social support, acculturation, current mental health status and stigma. Eleven demographic items were created to record information on marital status (single, never married; unmarried in a relationship; married; divorced or widowed), age (in years), gender (male/female), length of residence in the UK (months), education (nursery school to 8th grade; college without ‘matura’ (equivalent of A levels); college with A-levels; higher degree, recoded for analysis as ‘no higher degree’/‘higher degree’), family structure (number of children, residence of the children in Poland or in the UK or both if more than one child, residence of the partner in Poland or in the UK; not having a partner or having a partner in the outside of the UK was combined into the same category), employment status (full-time; part-time; unemployed; student; retired). Knowledge of the NHS and mental health services was assessed using three items: if participants knew how they, their friends or family members could access any NHS services; if participants knew how to access mental health services, and; to name any mental health services they were aware of in the UK. The first two items were rated on a 4-point Likert Scale ranging from 1 (‘not at all’) to 4 (‘very well’), and summed up to indicate knowledge of services. The scale was created by the author and was not based on a validated measure. We assessed language proficiency using four items in regard to writing, reading, speaking and comprehending English language (e.g. ‘My writing skills in English are’). Participants responded on a 5-point Likert scale ranging from 1 (‘very bad’) to 5 (‘very good’). Thus, the total score ranged from 4 to 20, with higher score indicated better language proficiency.

Mental health status was assessed using the General Health Questionnaire 12 (GHQ-12) [24], a twelve-item scale intended to screen for general (non-psychotic) psychiatric morbidity. The Polish version used here was derived from the scale with 30 items that had been translated into Polish showing strong reliability and validity in a clinical population, but its specificity and sensitivity in ‘caseness’ detection had not been assessed [25]. The measure with 12 items was validated both in general and clinical populations in non-Polish populations, with strong psychometric and clinical properties [26, 27]. All items have four responses, from ‘better than usual’ to ‘much less than usual’. We used the GHQ scoring method (0–0–1-1), as widely recommended [24]; Scores were summed by adding all items on the scale ranging from 0 to 12. A score of 4 or more has been used as a cut-off point for ‘caseness’ [28–30].

Perceived social support was measured using the Multidimensional Scale of Perceived Social Support (MSPSS) [31], which consists of 12 items designed to measure perceived social support from family, friends and a significant other. The scale has excellent internal consistency [32], factorial validity and construct validity [32, 33]. Response options range from 1 (‘very strongly disagree’) to 7 (‘very strongly agree’), giving a total score between 7 and 84. In the current study Polish validated version of the survey was used, which also showed robust psychometric properties [34].

Acculturation was assessed via an adapted version of the East Asian Acculturation Measure (EAAM) [35], where ‘Asian’ and ‘American’ were substituted with ‘Polish’ and ‘British’, respectively. We used a 7-items subscale of the instrument assessing separation, considered the opposite of acculturation [35]. Example items included ‘I prefer going to social gatherings where most people are Polish’. Items were assessed on a Likert scale from 1 (‘strongly disagree’) to 7 (‘strongly agree’). The total score was derived by summing all items, where higher scores indicated greater separation. The subscale of the original instrument yielded strong inter-rater reliability [35], and the Polish version has previously been used amongst nurses working in the UK (Występowanie szoku kulturowego i strategii akulturacji wśród polskich pielęgniarek i pielęgniarzy w Wielkiej Brytanii, unpublished).

Help-seeking self-stigma was assessed using the Self-Stigma of Seeking Help (SSOSH) [36], a 10-item tool rated on a 5-point scale ranging from 1 (‘strongly disagree’) to 5 (‘strongly agree’). The total score was obtained by summing reverse- and positive-scored items, for example ‘It would make me feel inferior to ask a therapist for help’, where the higher score indicates greater help-seeking self-stigma. The total score for the measure ranges from 10 to 50, with higher score reflecting greater self-stigma. The measure had good psychometric properties [36].

Except for categorical demographic variables, as described, all other measures were treated as continuous variables. We found strong internal consistency for all measures (Cronbach’s alpha ranging 0.80–0.94) [37, 38].

### Missing data

The MCAR Little’s test was conducted on the final (*n* = 536) to determine whether the data were missing completely at random (MCAR), thus if complete-case analysis could be used (*n* = 536) (Little, 1988). We used multiple imputation to recover missing data on predictors and outcomes, performed at the item-level as it has been found to produce more robust estimates [39]. We used the default method within SPSS, which scans the data and automatically chooses either the monotone method or fully conditional specification depending on the pattern of missing values [40]. All available predictors and outcome variables contributed to predict missing data in the predictors, producing 20 datasets [41]. In addition, we imputed missing values on single items within the outcome – intensions of future help-seeking. We constrained imputed variables to be scale-consistent and rounded to the nearest integer.

### Statistical analysis

First, demographic characteristics of the non-imputed sample (*n* = 536) were explored using frequency tables, and compared with the distribution of these characteristics in the general Polish population in the UK – to assess representativeness of our sample – using Z-tests. Comparisons between men and women were also made as sex was one of the hypothesised predictors of help-seeking due to differences in symptomology between both genders – potentially resulting in different help-seeking patterns [42]. We compared demographic data of our sample with those compiled by the Migration Research Unit at University College London and the ESRC Centre for Population Change, which were based on the Worker Registration Scheme, National insurance Number registrations, International Passenger Survey, and the Labour Force Survey [43]. Also, data from Accession Monitoring Report was used [44]. The data represents period between 2009 and 2011, the closest available population data to the study period.

Unadjusted and adjusted linear regression models were used to assess the association between predictors and the future intentions of mental health help-seeking. In adjusted models, predictors were added sequentially in order of the strength of association with the outcome from univariable analyses. Variables which led to a significant change in the total variance explained by the model based on the r-squared change test (*p* ≤ 0.05) were retained in the model. As the distribution of the GHSQ was highly positively skewed, the analyses were rerun with the logged version of the outcome (results not shown): producing findings consistent with those obtained with the unlogged total score, which we present here as they are easier to interpret.

For past utilisation of mental health services, we used unadjusted and adjusted logistic regression models following the same model building process. All analyses were conducted in IBM SPSS 22 for Windows [40]. Regression analyses were performed on the multiply-imputed sample, with each imputation analysed separately and subsequently pooling results using an automatic procedure within SPSS [40]. Sensitivity analyses including complete-cases only were conducted following the same procedure, and presented as supplemental material (Additional file 1: Tables S2 & S3).

## Results

### Missing data

Out of 536 participants, 18.5% (*n* = 99) had some missing data (Additional file 1: Table S4); nevertheless, the total amount of missing data in this sample was around 1% across all items. The data within the final sample were not missing completely at random (Little’s test: χ2 = 2329.63, df = 2170; *p* = 0.009), thus requiring multiple imputation under the assumption data were missing at random (MAR).

### Sample characteristics

Most participants lived in England (74.6%) or Scotland (21.5%), with fewer from elsewhere in the UK (Table 2). Median length of stay in the UK was 14 months (IQR = 5.8–25.3). Most participants were either married (30.0%, *n* = 161) or in a stable relationship (35.3%, *n* = 189). Only 7 individuals (1.3%) indicated that their partner was not present in the UK. Nearly one-third of respondents had A levels (30.2%, *n* = 162) and 4.9% (*n* = 26) finished their education at the level of middle school. Most participants were employed (77.6%, *n* = 415), with men being more likely to be employed full-time (77.0% vs 55.5%; z = 4.15, *p* < 0.001). A greater proportion of women were employed part-time (18.7% vs 6.3%; z = − 4.00, *p* < 0.001) or unemployed (15.0% vs 3.5%; z = − 4.14, *p* < 0.001).

**Table 2:** Demographic characteristics of the sample

Characteristics	Men (*n* = 174)^a^	Women (*n* = 353)^a^	Total (*N* = 536)
	*n* (%)	*n* (%)	*n* (%)
Marital Status			
In relationship (unmarried)	59 (33.9)	127 (36.0)	189 (35.3)
Married	38 (21.8)	120 (34.0)	161 (30.0)
Single, never married	64 (36.8)	64 (18.1)	129 (24.1)
Divorced	13 (7.5)	37 (10.5)	50 (9.3)
Widowed	0 (0.0)	4 (1.1)	4 (0.7)
Missing	0 (0.0)	1 (0.3)	3 (0.6)
Living Place			
England	134 (77.0)	262 (74.2)	400 (74.6)
Scotland	35 (20.1)	76 (21.5)	112 (20.9)
Wales	3 (1.7)	11 (3.1)	15 (2.8)
Northern Ireland	1 (0.6)	2 (0.6)	4 (0.7)
Missing	1 (0.6)	2 (0.6)	5 (0.9)
Education			
Nursery school to 8th grade	11 (6.3)	15 (4.3)	26 (4.9)
College without A Levels	57 (32.8)	102 (28.9)	162 (30.2)
College with A Levels	41 (23.6)	78 (22.1)	122 (22.8)
BA/BSc	29 (16.7)	64 (18.1)	94 (17.5)
MA/MSc	32 (18.4)	83 (23.5)	115 (21.5)
PhD	3 (1.7)	8 (2.3)	11 (2.1)
Missing	1 (0.6)	3 (0.9)	6 (1.1)
Employment Status			
Employed full-time	134 (77.0)	196 (55.5)	336 (62.7)
(≥ 35 h a week)			
Employed part-time	11 (6.3)	66 (18.7)	79 (14.9)
(≤ 20 h a week)			
Unemployed	6 (3.5)	53 (15.0)	59 (11.0)
Student	21 (12.1)	36 (10.2)	57 (10.6)
Retired	1 (0.6)	0 (0.0)	1 (0.2)
Missing	1 (0.6)	2 (0.6)	3 (0.6)
Partner not living in the UK	6 (3.5)	1 (0.3)	7 (1.3)
Length of stay in the UK (in months)			
< 3	29 (16.7)	55 (15.6)	82 (15.3)
≥ 3 < 12	55 (31.6)	109 (30.9)	166 (31.0)
≥ 12 < 24	43 (24.7)	86 (24.4)	129 (24.1)
≥ 24	47 (27.0)	103 (29.2)	153 (28.5)
Missing	0 (0.0)	0 (0.0)	6 (1.1)
	Median (range)	Median (range)	Median (range)
Age	30.5 (18–64)	31 (18–62)	31 (18–64)
Number of children	1 (0–5)	0 (0–4)	0 (0–5)

*Note. BA/BSc* Bachelor of Arts/Bachelor of Science, *MA/MSc* Master of Arts/Master of Science, *PhD* Doctor of Philosophy, *UK* United Kingdom

^a^*n* = 9 did not indicate their gender

Compared with the Polish population in the UK, our study sample had a greater proportion of females (65.9% vs 51.0%; z = 6.9, *p* < 0.001), participants aged between 20 and 34 (63.8% vs 57.3%; z = 3.0, *p* = 0.002), people educated to degree level (41% vs 23%; z = 9.9, *p* < 0.001), people who were unemployed (11% vs 3.5%; z = 9.4, *p* < 0.001) and people with dependants living in the UK (35.8% vs 12.0%; z = 16.9, *p* < 0.001). Our participants were less likely to be aged over 60 (0.7% vs 4.6%; z = 4.3, *p* < 0.001).

### Mental health and help-seeking outcomes

Over half the sample met GHQ-12 criteria indicating a likely mental health disorder (58.4%). Nonetheless, only 16% of participants (*n* = 84) had sought professional mental health help during their stay in the UK (Table 3), with a comparable (z = 1.46, *p* = 0.13) proportion of women (17.6%; *n* = 62) and men (12.6%; *n* = 22). The most common source of help was a psychologist/psychotherapist (25%, *n* = 21), GP (16%, *n* = 13) or counsellor (5%, *n* = 4); 13% of participants (*n* = 11) did not know the profession of their health provider. Participants were also asked about awareness of mental health services. A total of 297 participants responded to this question (55.4%), with the most common responses listed as GP or NHS in general (37.7%, *n* = 112), charities (28.6%, *n* = 85) or specific NHS mental health services (4.7%, *n* = 14).

**Table 3:** Descriptive statistics of exposure and outcome variables

	Total (*N* = 536)	Men (*n* = 174)	Women (*n* = 353)	
Variable	*n* (%)	*n* (%)	*n* (%)	*p* for difference
GHQ (Cases)^a^	313 (58.4)	97 (55.8)	216 (61.2)	0.21
GHSQ (Past)	84 (15.7)	22 (12.6)	62 (17.6)	0.26
	Median (IQR)	Median (IQR)	Median (IQR)	*p*-value
GHSQ (Intentions)	10.00 (8.00)	10.00 (9.00)	11.00 (8.00)	0.57
	Mean (SD)	Mean (SD)	Mean (SD)	*p*-value
SSOSH	24.65 (6.39)	26.11 (6.41)	23.93 (6.26)	< 0.001
MSPSS	42.33 (10.71)	40.90 (10.82)	43.04 (10.56)	0.03
EAAM^b^	22.22 (8.37)	23.68 (7.97)	21.50 (8.46)	0.05
English language proficiency	15.32 (3.88)	14.96 (3.86)	15.50 (3.88)	0.14
Knowledge of NHS	4.21 (1.79)	3.96 (1.66)	4.34 (1.85)	0.02

*Note. GHQ* the General Health Questionnaire, *GHSQ* the General Health Seeking Questionnare, *IQR* Interquartile Range, *SD* Standard Deviation, *SSOSH* the Self-Stigma of Seeking Help, *MSPSS* the Multidimensional Scale of Perceived Social Support, *EAAM* the East Asian Acculturation Measure, *NHS* the National Health Services

^a^GHQ ≥ 4 was considered as a case

^b^Higher score indicated lower acculturation (higher separation)

Participants on average rated the likelihood of seeking professional help in the UK in the next 3 months as 10.00 (IQR = 8.00), on a scale ranging from 5 to 35. The most likely source of help, on a scale from 1 to 7, was family doctor/GP (Mean = 3.40; SD = 2.05), followed by mental health professionals (Mean = 2.63, SD = 1.95), with other, not listed, professionals (M = 2.29, SD = 1.69), charities (Mean = 1.83; SD = 1.47) and phone help-lines (Mean = 1.68, SD = 1.22) being less likely source of professional help.

### Predictors of future intentions of mental health help-seeking

In our final model (Table 4), the intention of help-seeking increased by 0.15 unit for each year of age (0.09–0.21, *p* = 0.001), not having a partner at all or not having a partner living in the UK, as opposed to having a partner living in the UK, was associated with 1.17 unit increase (B = 1.17, 95%CI: 0.19–2.16, *p* = 0.02) (all predisposing factors), history of past help-seeking behaviours (B = 2.13, 95% CI: 0.73–3.52, *p* = 0.003), greater knowledge of the NHS (B = 0.32, 95% CI: 0.03–0.62, *p* = 0.03) (both enabling factors) and worse mental health as rated with GHQ-12 (B = 0.54, 95% CI: 0.42–0.65, *p* < 0.001) (need factor) were associated with a greater intentions of future help-seeking for mental health problems. Greater perceived stigma to seek help for mental health problems (B = − 0.19, 95% CI: -0.27 to − 0.11, *p* < 0.001) (predisposing factor) and education to undergraduate level or above (B = − 1.25, 95% CI: -2.24 to − 0.25, *p* < 0.001) (enabling factor) reduced intentions to seek help in the future.

**Table 4:** Predictors of intentions of future help-seeking both in unadjusted and adjusted linear regression

	Unadjusted			Adjusted^a^		
	Β	95% Cl	*p*-value	Β	95% Cl	*p*-value
Constant				10.19	7.01 to 13.38	0.001
Predisposing variables						
Age	0.15	0.09 to 0.21	0.001	0.09	0.04 to 0.15	0.001
Gender						
Female (reference category)						
Male	−0.34	−1.49 to - 0.82	0.57	0.34	−0.70 to 1.38	0.52
Marital status						
Single, never married (reference category)						
In a relationship	−1.05	−2.46 to 0.35	0.14	−1.38	−5.76 to 3.01	0.54
Married	0.12	−1.34 to 1.57	0.87	−1.30	−5.68 to 3.09	0.56
Divorced/widowed	3.02	1.04 to 5.00	0.003	−0.02	−1.93 to 1.89	0.99
Length of stay in the UK (in months)						
< 3 (reference category)						
≥ 3 < 12	−0.46	−2.14 to 1.23	0.59	−0.62	−2.09 to 0.85	0.41
≥ 12 < 24	0.71	−1.05 to 2.47	0.43	−0.51	−2.09 to 1.07	0.53
≥ 24	−0.24	−1.94 to 1.47	0.79	−1.29	−2.89 to 0.32	0.12
Children in the UK						
Yes (reference category)						
No/Not all	0.53	−1.28 to 2.34	0.57	−0.37	−1.58 to .85	0.55
Not applicable	−1.67	−2.84 to −0.50	0.005	−0.16	−1.88 to 1.56	0.86
Partner present in the UK						
Yes (reference category)						
No/Not applicable	1.43	0.30 to 2.55	0.013	1.17	0.19 to 2.16	0.02
Help-seeking self-stigma	−0.20	−0.28 to − 0.12	0.001	− 0.19	−0.27 to − 0.11	< 0.001
Enabling variables						
Education						
Nursery schools to 8th grade/college (reference category)						
Graduate/postgraduate	−1.76	−2.85 to −0.67	0.002	−1.25	− 2.24 to − 0.25	0.01
Job Status						
Employed part-time (reference category)						
Employed full-time	−1.24	−2.79 to .31	0.12	−0.51	−1.88 to 0.85	0.46
Unemployed	−0.55	−2.68 to 1.59	0.62	−1.36	−3.25 to 0.54	0.16
Retired	5.62	−3.75 to 14.98	0.24	5.36	−4.03 to 14.74	0.26
Student	−2.81	−4.96 to −0.65	0.01	−1.08	− 3.07 to 0.90	0.29
Past help-seeking behaviours						
No (reference category)						
Yes	4.14	2.72 to 5.57	0.001	2.13	0.73 to 3.52	0.003
Knowledge of NHS	0.12	−0.19 to 0.42	0.45	0.32	0.03 to 0.62	0.03
English proficiency	−0.30	−0.44 to − 0.16	0.001	−0.14	− 0.30 to 0.02	0.08
Acculturation	0.04	−0.02 to 0.11	0.20	0.02	−0.04 to 0.08	0.51
Perceived Social Support	−0.14	−0.19 to − 0.09	0.001	−0.01	− 0.07 to 0.04	0.60
Need variable						
Mental health status	0.54	0.43 to 0.65	0.001	0.54	0.42 to 0.65	< 0.001

^a^Adjusted for age, presence of partner in the UK, help-seeking self-stigma, education, past help-seeking behaviours, knowledge of NHS and mental health status

### Predictors of past help-seeking behaviours

In our final model (Table 5), older age (OR = 1.07, 95% CI: 1.02–1.11, *p* = 0.003), being in the UK for over 2 years (OR = 4.90, 95% CI: 1.52–15.80, *p* = 0.008), not having children (OR = 2.58, 95% CI: 1.33–5.03, *p* = 0.005) (all predisposing factors), having greater knowledge of the NHS (OR = 1.07, 95% CI: 1.02–1.11, *p* = 0.003) (enabling factor) and worse mental health (OR = 1.11, 95% CI: 1.04–1.20, *p* = 0.004) (need factor) were associated with greater odds of past help-seeking behaviours for mental health problems. In contrast, having greater help-seeking self-stigma (OR = 0.85, 95% CI: 0.81–0.90, *p* < 0.001) (predisposing factor) and greater perceived social support (OR = 0.97, 95% CI: 0.94–1.00, *p* = 0.04) (enabling factor) were associated with lower odds of past help-seeking behaviours.

**Table 5:** Predictors of past help-seeking behaviours both in unadjusted and adjusted logistic regression

	Unadjusted			Adjusted^a^		
	OR	95% Cl	*p*-value	OR	95% Cl	*p*-value
Constant				0.08	0.004 to 1.57	0.1
Predisposing variables						
Age	1.04	1.01 to 1.06	0.006	1.07	1.02 to 1.11	0.003
Gender						
Female (reference category)						
Male	0.68	0.40 to 1.16	0.16	0.81	0.42 to 1.55	0.52
Marital status						
Single, never married (reference category)						
In a relationship	1.17	0.59 to 2.31	0.66	1.47	0.65 to 3.35	0.35
Married	1.75	0.90 to 3.41	0.10	2.11	0.85 to 5.24	0.11
Divorced/widowed	2.49	1.10 to 5.64	0.03	2.87	0.95 to 8.65	0.06
Length of stay in the UK (in months)						
< 3 (reference category)						
≥ 3 < 12	1.91	0.61 to 5.94	0.27	1.69	0.49 to 5.82	0.40
≥ 12 < 24	4.10	1.36 to 12.35	0.01	3.00	0.91 to 9.94	0.07
≥ 24	6.59	2.27 to 19.13	0.001	4.90	1.52 to 15.80	0.008
Children in the UK						
Yes (reference category)						
No/Not all	0.40	0.14 to 1.15	0.09	0.22	0.06 to 0.78	0.02
Not applicable	1.12	0.69 to 1.84	0.65	2.58	1.33 to 5.03	0.005
Partner present in the UK						
Yes (reference category)						
No/Not applicable	0.99	0.61 to 1.60	0.96	0.93	0.51 to 1.71	0.81
Help-seeking self-stigma	0.87	0.83 to 0.91	0.001	0.85	0.81 to 0.90	< 0.001
Enabling variables						
Education						
Nursery schools to 8th grade/college (reference category)						
Graduate/postgraduate	1.22	0.72 to 2.09	0.46	0.89	0.47 to 1.71	0.74
Job Status						
Employed part-time (reference category)						
Employed full-time	0.93	0.66 to 1.30	0.83	0.78	0.36 to 1.69	0.53
Unemployed	0.70	0.52 to 3.03	0.48	0.68	0.21 to 2.15	0.51
Retired^b^						
Student	1.26	0.53 to 3.10	0.61	0.76	0.24 to 2.38	0.64
Knowledge of NHS	1.45	1.31 to 1.62	< 0.001	1.41	1.20 to 1.66	< 0.001
English proficiency	1.06	1.02 to 1.09	0.09	0.97	0.88 to 1.07	0.58
Acculturation	0.96	0.93 to 0.99	0.003	0.98	0.94 to 1.01	0.22
Perceived Social Support	0.98	0.96 to 1.00	0.06	0.97	0.94 to 1.00	0.04
Need variable						
Mental health status	1.05	1.00 to 1.11	0.07	1.11	1.04 to 1.20	0.004

*Note. OR* odds ratio

^a^Adjusted for age, length of stay in the UK, presence of children in the UK, help-seeking self-stigma, knowledge of NHS, perceived social support and mental health status

^b^Estimates not produced due to a low number of participants (*n* = 21)

### Complete cases analyses

There were some differences found between adjusted linear regression conducted with the multiply imputed sample and complete cases only (see Additional file 1: Tables S2 & S3). In the complete case analysis, being in the UK for 24 months or longer was associated with lower intentions of future help-seeking compared to being in the UK for 3 months or less (B = − 1.88, − 3.57 to − 0.19, *p* = 0.03), such an association was not found in the main analysis. On the other hand, having a partner in the UK (B = 0.91, 0.14 to 1.95, *p* = 0.09) and knowledge of NHS (B = 0.14, − 0.16 to 0.44, *p* = 0.37) were not associated with intentions of help-seeking in the complete case analysis, whereas this association was shown by the analysis with the imputed sample.

## Discussion

In this cross-sectional survey of Polish immigrants living in the UK, we found strong evidence that older age and living circumstances (predisposing factors), as well as knowledge of the NHS, social support, mental health stigma and education (enabling factors) were associated with aspects of past and future help-seeking for mental health problems. Finally, as commonly found in the literature, current mental health status was associated with both past help-seeking and intentions [18]. In the next section, we discuss the role of predisposing and enabling factors in more detail.

### Predisposing factors

Older age has been consistently associated with greater intensions of help-seeking and higher odds of past help-seeking, including amongst relatively young migrants [45]. As shown by qualitative studies, stigma related to mental health is still deep-rooted among Polish people living abroad, possibly reflecting people’s attitudes in their home country [7, 16, 46]. Here, we found strong associations between self-stigma and past and future intentions to seek help for mental health problems. Notably, these associations were not attenuated by other variables included in our models, particularly acculturation, length of stay in the UK, education or previous help-seeking behaviours, which would be expected to have confounding effects on stigma [47].

Being single, or having a partner who did not live in the UK predisposed people to having greater intentions of future help-seeking. This may be partially explained by protective factors associated with marriage and cohabiting for mental health [48, 49]. Furthermore, participants who did not live with their children in the UK (compared with participants living with their children) had lower odds of past help-seeking, suggesting that strong family networks and support may encourage use of mental health services. Nonetheless, living with/without children was not associated with intensions of future help-seeking, which may suggest that the direction of this association is specific to past mental health seeking behaviours enabling stronger family networks, rather than family support being a predictor of mental health help-seeking. Finally, length of stay in the UK was found to predict past help-seeking behaviours. This may be associated with a greater awareness of available services, thus facilitating help-seeking. Consistent with this possibility, we found independent effects of knowledge of the NHS on both past and future intention of mental health help-seeking.

Contrary to previous research [18], gender was not associated with the outcome variables. This may have been due to the characteristics of the sample. Namely, women, who were hypothesised to be more likely to seek help, were more likely to have a degree, which in turn was associated with lower likelihood of help-seeking. Thus, the effect of gender, which is likely to predispose to help-seeking, may have been ‘cancelled out’ by education that was found to have a protective effect.

### Enabling factors

Greater knowledge of NHS was associated both with an increase in intentions of help-seeking and with presence of past help-seeking behaviours. As reported by previous qualitative studies with Polish population in the UK, poor knowledge of NHS is associated with other factors, such as low acculturation and past (negative) experience of the health services [7, 16]. We adjusted for these variables, which did not confound our observations. Greater perceived social support was linked with lower odds of past help-seeking. This may be because social support is protective for mental health. Alternatively, family may be the first source of help, as found in previous studies [23].

Having a degree was associated with lower intentions of help-seeking, possibly having a protective effect [18]. Past help-seeking behaviours also had enabling effect on intentions of future help-seeking. These effects were attenuated, though remained significant, after accounting for other confounders including mental health status as measured via the GHQ-12, suggesting that prolonged mental health difficulties cannot exclusively explain the link between the past help-seeking behaviours and intentions of help-seeking. Past help-seeking behaviours may have resulted in reduced self-stigma [50], although our cross-sectional study design precludes us from assessing the direction of these effects. Acculturation, language proficiency were not linked to either of the outcomes in our study, as also found by previous research [45].

### Limitations/strengths and future research

One of the main limitations of our study is that there were discrepancies in the results using the multiply imputed sample and complete cases only. It is problematic to determine which set of values provided more accurate estimate of associations. We did find some evidence that data in our study were more likely to be missing at random rather than completely at random, however the distinction between data missing at random (MAR) and not at random (MNAR) could not be determined. Despite certain discrepancies between the analyses, the majority of estimates were consistent thus their robustness can be assumed. Moreover, some of the measures used in the study were not available in Polish language, thus they were back-translated from an English version. Due to lack of resources they were not validated prior to the use in the study. Nonetheless, they were a subject to a rigorous process of back-translation according to commonly acknowledged practice guidelines [23]. Also, as indicated by Cronbach’s alpha they were found to have high internal consistency.

Finally, participants were recruited through social and online news media due to convenience of such methodology. Hence, our sample may be more representative to younger individuals as those are more commonly users of social media or Polish online groups. Possibly due to volunteer bias, females and individuals with a degree were also more likely to participate in our survey. Most strikingly, our sample comprises a large proportion of people who may potentially suffer from mental health problems. This may simply reflect a greater interest in the study among those who experience poor mental health. Although our study cannot claim to be representative for the general Polish population, it captures a large number of young, well-educated people who may be particularly vulnerable to mental health problems. As they volunteered to our study, they have interest in mental health, whilst revealing low intentions of seeking help. Hence, further research is needed, with a larger number of participants – ideally randomly chosen – which would be more generalisable for the Polish, and Eastern European in general, population in the UK.

## Conclusions

The participants included in our study had particularly poor mental health, whilst exhibiting low intentions of help-seeking, which emphasises the need to reach out to these individuals. Due to large numbers of Eastern European migrants in the UK, it is vital to ensure that these populations receive adequate mental health support. Hence, it is crucial to tailor interventions provided to specific groups of Polish, or migrant, populations acting on mutable factors such as knowledge of NHS or help-seeking self-stigma. For instance, parents living with their children in the UK are more likely to seek help than individuals whose children do not live in the UK. Thus, those with families may be already more actively searching for information about services and it needs to be ensured that this information is easily available. Whereas, Polish migrants living in the UK on their own and facing mental health difficulties are more reluctant to seek help. Hence, more active effort ought to be made to reach these individuals.

## Additional file

Additional file 1: **Table S1.** Exposure and outcome variables measured by aggregated scales with associated measures. **Table S2.** Predictors of intentions of future help-seeking both in unadjusted and adjusted linear regression conducted using complete cases only. **Table S3.** Predictors of past help-seeking behaviours both in unadjusted and adjusted logistic regression conducted with complete cases only. **Table S4.** The proportions of cases with all items and at least one item missing in the total and final samples. (DOC 163 kb)

## Abbreviations

EAAM: East Asian Acculturation Measure
GHQ: General Health Questionnaire
GHSQ: General Help Seeking Questionnaire
MAR: Missing at random
MCAR: Missing completely at random
MNAR: Missing not at random
MSPSS: Multidimensional Scale of Perceived Social Support
NHS: National Health Service
SSOSH: Self-Stigma of Seeking Help
UK: United Kingdom
