# Supplementary material for: Predictors of mental health help-seeking among polish people living the United Kingdom
Source: BMC Health Serv Res. 2018 Sep 6;18:693. doi: 10.1186/s12913-018-3504-0 (PMC6127920; doi:10.1186/s12913-018-3504-0)
Supplement: Supplementary file 1 — Table S1. Exposure and outcome variables measured by aggregated scales with associated measures. Table S2. Predictors of intentions of future help-seeking both in unadjusted and adjusted linear regression conducted using complete cases only. Table S3. Predictors of past help-seeking behaviours both in unadjusted and adjusted logistic regression conducted with complete cases only. Table S4. The proportions of cases with all items and at least one item missing in the total and final samples. (DOC 163 kb) [file 12913_2018_3504_MOESM1_ESM.doc]

| **Additional file 1: Table S1** Exposure and outcome variables measured by aggregated scales with associated measures. | |  |  |
| --- | --- | --- | --- |
|  | **Measure** | Source of the Polish version obtained | Internal consistency in the current study (Cronbach`s alpha) |
| **Outcome variable** |  |  |  |
| Intentions of help-seeking | The General Help Seeking Questionnaire (GHSQ; Wilson, Deane, Ciarrochi & Rickwood, 2005) | Back-translated | 0.80 |
| **Exposure variables** |  |  |  |
| Acculturation | The Separation subscale of the East Asian Acculturation Measure (EAAM; Barry, 2001) | Cholewa & Majda (unpublished) | 0.82 |
| Help-seeking self-stigma | The Self-Stigma of Seeking Help (SSOSH, Vogel, Wade, & Haake, 2006) | Self-translated | 0.80 |
| Perceived social support | The Multidimensional Scale of Perceived Social Support (MSPSS; Zimet, Dahlem, Zimet, & Farley, 1988) | Adamczyk (2013) | 0.94 |
| Mental health status | The 12-item General Health Questionnaire (GHQ-12; Goldberg & Williams, 1988) | Fryderycka et al. (2010) | 0.93 |
| Knowledge of NHS | Miscellaneous | Created in Polish | 0.81 |
| English language proficiency | Miscellaneous | Created in Polish | 0.94 |
|  | | | |

| **Table S2** Predictors of intentions of future help-seeking both in unadjusted and adjusted linear regression conducted using **complete cases only**. | | | | | | | |
| --- | --- | --- | --- | --- | --- | --- | --- |
|  | Unadjusted | | |  | Adjusted† | | |
|  | Β | 95% Cl | P-value |  | Β | 95% Cl | P-value |
| Constant |  |  |  |  | 11.05 | 7.69 to 14.41 | <0.001 |
| **Predisposing variables** |  |  |  |  |  |  |  |
| Age | 0.14 | 0.08 to 0.21 | <0.001 |  | **0.08** | **0.02 to 0.14** | **0.006** |
| Gender |  |  |  |  |  |  |  |
| Female (reference category) | | | |  |  |  |  |
| Male | -0.61 | -1.83 to 0.61 | 0.32 |  | 0.14 | -0.95 to 1.23 | 0.78 |
| Marital status |  |  |  |  |  |  |  |
| Single, never married (reference category) | | | |  |  |  |  |
| In a relationship | -0.67 | -2.17 to 0.82 | 0.37 |  | -0.29 | -4.71 to 4.14 | 0.90 |
| Married | 0.66 | -0.91 to 2.23 | 0.41 |  | -0.04 | -4.49 to 4.40 | 0.90 |
| Divorced/widowed | 3.39 | 1.18 to 5.60 | 0.003 |  | 0.43 | -1.65 to 2.52 | 0.68 |
| Length of stay in the UK (in months) | | |  |  |  |  |  |
| < 3 (reference category) | | | |  |  |  |  |
| ≥ 3 < 12 | -0.98 | -2.79 to 0.83 | 0.29 |  | -0.87 | -2.43 to 0.69 | 0.28 |
| ≥ 12 < 24 | 0.07 | -1.77 to 1.90 | 0.94 |  | -0.89 | -2.50 to 0.73 | 0.28 |
| ≥ 24 | -1.12 | -2.95 to 0.70 | 0.23 |  | **-1.88** | **-3.57 to -0.19** | **0.03** |
| Children in the UK |  |  |  |  |  |  |  |
| Yes (reference category) | | | |  |  |  |  |
| No/Not all | -1.72 | -2.95 to -0.50 | 0.006 |  | -0.76 | -2.02 to 0.50 | 0.24 |
| Not applicable | 0.32 | -1.73 to 2.37 | 0.76 |  | -0.35 | -2.25 to 1.55 | 0.72 |
| Partner in the UK |  |  |  |  |  |  |  |
| Yes (reference category) | | | |  |  |  |  |
| No/Not applicable | 1.12 | 2.33 to 0.09 | 0.07 |  | 0.91 | 0.14 to 1.95 | 0.09 |
| Help-seeking self-stigma | -0.22 | -.309 -.137 | <0.001 |  | **-0.19** | **-0.28 to -0.11** | **<0.001** |

| **Table S2** (Continued) | | | | | | | |
| --- | --- | --- | --- | --- | --- | --- | --- |
|  | Unadjusted | | |  | Adjusted† | | |
|  | Β | 95% Cl | p-value |  | Β | 95% Cl | p-value |
| **Enabling variables** |  |  |  |  |  |  |  |
| Education |  |  |  |  |  |  |  |
| Nursery schools to 8^th^ grade/college (reference category) | | | |  |  |  |  |
| Graduate/postgraduate | -1.64 | -2.79 to -0.48 | 0.005 |  | **-1.08** | **-2.11 to -0.06** | **0.04** |
| Job Status |  |  |  |  |  |  |  |
| Employed part-time |  |  |  |  |  |  |  |
| Employed full-time | -1.56 | -3.19 to 0.08 | 0.06 |  | -0.72 | -2.15 to 0.71 | 0.32 |
| Unemployed | -0.70 | -2.99 to 1.60 | 0.55 |  | -0.98 | -2.99 to 1.04 | 0.34 |
| Retired | 7.96 | -4.07 to 19.98 | 0.19 |  | 10.53 | -0.004 to 21.06 | 0.05 |
| Student | -2.83 | -5.02 to -0.62 | 0.01 |  | -0.86 | -2.87 to 1.16 | 0.40 |
| Past help-seeking behaviours | | | |  |  |  |  |
| No |  |  |  |  |  |  |  |
| Yes | 4.68 | 3.18 to 6.19 | <0.001 |  | **2.77** | **1.30 to 4.24** | **<0.001** |
| Knowledge of NHS | -0.03 | -0.35 to 0.29 | 0.86 |  | 0.14 | -0.16 to 0.44 | 0.37 |
| English language proficiency | -0.32 | -0.46 to -0.17 | <0.001 |  | -0.14 | -0.31 to 0.02 | 0.09 |
| Acculturation | 0.05 | -0.02 to .12 | 0.18 |  | 0.02 | -0.05 to 0.08 | 0.56 |
| Perceived Social Support | -0.15 | -0.21 to -0.10 | <0.001 |  | -0.04 | -0.10 to 0.02 | 0.15 |
| **Need variable** |  |  |  |  |  |  |  |
| Mental health status | 0.54 | 0.42 to 0.66 | <0.001 |  | **0.52** | **0.39 to 0.64** | **<0.001** |
| † Adjusted for age, presence of partner in the UK, help-seeking self-stigma, education, past help-seeking behaviours, knowledge of NHS and mental health status. | | | | | | | |

| **Table S3** Predictors of past help-seeking behaviours both in unadjusted and adjusted logistic regression conducted with **complete cases only**. | | | | | | | |
| --- | --- | --- | --- | --- | --- | --- | --- |
|  | Unadjusted | | |  | Adjusted† | | |
|  | OR | 95% Cl | P-value |  | OR | 95% Cl | P-value |
| Constant |  |  |  |  | 0.02 |  | 0.03 |
| **Predisposing variables** |  |  |  |  |  |  |  |
| Age | 1.03 | 1.00 to 1.06 | 0.047 |  | 1.08 | 1.03 to 1.14 | 0.002 |
| Gender |  |  |  |  |  |  |  |
| Female (reference category) | | | |  |  |  |  |
| Male | 0.70 | 0.39 to 1.23 | 0.21 |  | 0.88 | 0.43 to 1.80 | 0.73 |
| Marital status |  |  |  |  |  |  |  |
| Single, never married (reference category) | | | |  |  |  |  |
| In a relationship | 1.19 | 0.57 to 2.52 | 0.64 |  | 1.96 | 0.79 to 4.86 | 0.15 |
| Married | 1.68 | 0.80 to 3.55 | 0.17 |  | 2.73 | 0.97 to 7.63 | 0.056 |
| Divorced/widowed | 2.81 | 1.12 to 7.05 | 0.03 |  | 4.82 | 1.29 to 18.00 | 0.02 |
| Length of stay in the UK (in months) | | |  |  |  |  |  |
| < 3 (reference category) | | | |  |  |  |  |
| ≥ 3 < 12 | 1.78 | 0.56 to 5.69 | 0.33 |  | 1.57 | 0.42 to 5.81 | 0.50 |
| ≥ 12 < 24 | 3.57 | 1.18 to 10.86 | 0.03 |  | 2.46 | 0.71 to 8.59 | 0.16 |
| ≥ 24 | 5.31 | 1.79 to 15.78 | 0.003 |  | 3.69 | 1.06 to 12.86 | 0.04 |
| Children in the UK |  |  |  |  |  |  |  |
| Yes (reference category) | | | |  |  |  |  |
| No/Not all | 0.27 | 0.06 to 1.19 | 0.08 |  | 0.10 | 0.02 to 0.57 | 0.001 |
| Not applicable | 1.26 | 0.73 to 2.18 | 0.40 |  | 2.76 | 1.31 to 5.80 | 0.01 |
| Partner in the UK |  |  |  |  |  |  |  |
| Yes (reference category) | | | | | | | |
| No/Not applicable | 0.97 | 0.56 to 1.66 | 0.90 |  | 1.20 | 0.60 to 2.38 | 0.61 |
| Help-seeking self-stigma | 0.86 | 0.81 to 0.90 | <0.001 |  | 0.84 | 0.794 to 0.90 | <0.001 |

| **Table S3** (Continued) | | | | | | | |
| --- | --- | --- | --- | --- | --- | --- | --- |
|  | Unadjusted | | |  | Adjusted† | | |
|  | OR | 95% Cl | P-value |  | OR | 95% Cl | P-value |
| **Enabling variables** |  |  |  |  |  |  |  |
| Education |  |  |  |  |  |  |  |
| Nursery schools to 8^th^ grade/college (reference category) | | | |  |  |  |  |
| Graduate/postgraduate | 1.38 | 0.83 to 2.31 | 0.22 |  | 0.92 | 0.45 to 1.87 | 0.81 |
| Job Status |  |  |  |  |  |  |  |
| Employed part-time (reference category) | | | |  |  |  |  |
| Employed full-time | 0.87 | 0.43 to 1.76 | 0.70 |  | 0.70 | 0.30 to 1.62 | 0.40 |
| Unemployed | 0.43 | 0.13 to 1.43 | 0.17 |  | 0.33 | 0.08 to 1.39 | 0.13 |
| Retired†† |  |  |  |  |  |  |  |
| Student | 1.05 | 0.41 to 2.65 | 0.92 |  | 0.49 | 0.14 to 1.71 | 0.27 |
| Knowledge of NHS | 1.47 | 1.28 to 1.70 | <0.001 |  | 1.50 | 1.24 to 1.80 | <0.001 |
| English language proficiency | 1.07 | 1.00 to 1.15 | 0.047 |  | 1.00 | 0.89 to 1.11 | 0.94 |
| Acculturation | 0.95 | 0.92 to 0.98 | 0.002 |  | 0.98 | 0.94 to 1.02 | 0.26 |
| Perceived Social Support | 0.99 | 0.96 to 1.01 | 0.24 |  | 0.98 | 0.95 to 1.02 | 0.35 |
| **Need variable** |  |  |  |  |  |  |  |
| Mental health status | 1.06 | 1.00 to 1.12 | 0.045 |  | 1.15 | 1.06 to 1.25 | 0.001 |
| *Note.* OR = odds ratio.  † Adjusted for age, length of stay in the UK, presence of children in the UK, help-seeking self-stigma, knowledge of NHS, perceived social support and mental health status.  †† Estimates not produced due to a low number of participants (n = xx). | | | | | | | |

| **Table S4** The proportions of cases with all items and at least one item missing in the total and final samples. | | | | |
| --- | --- | --- | --- | --- |
| Variables | Total sample (N=671) | | Final sample (n=536) | |
|  | n (%) cases with **all** items missing | n (%) cases with **at least one** item missing | n (%) cases with **all** items missing | n (%) cases with **at least one** item missing |
| Marital Status† | 8 (1.2) | 8 (1.2) | 3 (0.6) | 3 (0.6) |
| Age† | 14 (2.1) | 14 (2.1) | 3 (0.6) | 3 (0.6) |
| Gender† | 15 (2.2) | 15 (2.2) | 9 (1.7) | 9 (1.7) |
| Length of stay in the UK† | 17 (2.5) | 17 (2.5) | 6 (1.1) | 6 (1.1) |
| Education† | 10 (1.5) | 10 (1.5) | 6 (1.1) | 6 (1.1) |
| Employment Status† | 8 (1.2) | 8 (1.2) | 4 (0.8) | 4 (0.8) |
| Children living in the UK† | 16 (2.4) | 16 (2.4) | 10 (1.9) | 10 (1.9) |
| Partner living in the UK† | 8 (1.2) | 8 (1.2) | 0 (0.0) | 0 (0.0) |
| Knowledge of NHS | 52 (7.8) | 55 (8.2) | 1 (0.2) | 4 (0.8) |
| English language proficiency | 52 (7.8) | 59 (8.8) | 0 (0.0) | 3 (0.6) |
| GHQ-12 | 70 (10.4) | 90 (8.9) | 0 (0.0) | 14 (2.6) |
| SSOSH | 104 (15.5) | 123 (18.3) | 0 (0.0) | 14 (2.6) |
| GHSQ (Intentions) †* | 129 (19.2) | 129 (19.2) | 0 (0.0) | 7 (1.3) |
| GHSQ (Past) * | 130 (19.4) | 141 (21.0) | 0 (0.0) | 0 (0.0) |
| EAAM | 127 (18.9) | 137 (20.4) | 3 (0.6) | 9 (1.7) |
| MSPSS | 135 (20.1) | 145 (21.6) | 9 (1.7) | 16 (3.0) |
| † Measured with only one item, thus n cases with all items missing equals n with at least one item missing.  * Outcome variables. | | | | |
